# Potential for a novel manganese porphyrin compound as adjuvant canine lymphoma therapy

**DOI:** 10.1007/s00280-017-3372-z

**Published:** 2017-07-06

**Authors:** M. K. Boss, M. W. Dewhirst, R. S. Sampaio, A. Bennett, A. Tovmasyan, K. G. Berman, A. W. Beaven, D. A. Rizzieri, I. Batinic-Haberle, M. L. Hauck, I. Spasojevic

**Affiliations:** 1 Department of Molecular Biomedical Sciences, North Carolina State University, Raleigh, NC 27607 USA; 2 Department of Environmental and Radiological Health Sciences, Colorado State University, Fort Collins, CO 80523 USA; 3 Department of Radiation Oncology, Duke University School of Medicine, Durham, NC 27710 USA; 4 Departamento de Quimica, CCEN, Universidade Federal da Paraiba, Joao Pessoa, PB Brazil; 5 Department of Clinical Sciences, North Carolina State University, Raleigh, NC 27607 USA; 6 Las Vegas Veterinary Specialty Center, Las Vegas, NV 89147 USA; 7 Bridge Pathology, Bristol, England, UK; 8 Department of Medicine, Duke University School of Medicine, Durham, NC 27710 USA; 9 Pharmaceutical Research Shared Resource, PK/PD Core Laboratory, Duke Cancer Institute, Durham, NC 27710 USA

**Keywords:** Manganese porphyrin, Canine, Lymph node, Lymphoma

## Abstract

**Purpose:**

Manganese porphyrins are redox-active drugs and superoxide dismutase mimics, which have been shown to chemosensitize lymphoma, a cancer which frequently occurs in dogs. This study aimed to identify critical information regarding the pharmacokinetics and toxicity of Mn(III) *meso*-tetrakis (*N*-n-butoxyetylpyridium-2-yl) porphyrin, (MnTnBuOE-2-PyP^5+^, MnBuOE) in dogs as a prelude to a clinical trial in canine lymphoma patients.

**Methods:**

A single-dose pharmacokinetic (PK) study in normal dogs was performed to determine the plasma half-life (*t*_1/2_) of MnBuOE. A dose reduction study was performed to establish the maximum tolerated dose (MTD) of MnBuOE. The safety and PK of a multi-dosing protocol was assessed.

**Results:**

Peak plasma drug concentration occurred 30 min post-injection. The *t*_1/2_ was defined as 7 h. MnBuOE induced an anaphylactic reaction and prolonged tachycardia. The MTD was defined as 0.25 mg/kg. The dogs were given MTD 3×/week for 2–3 weeks. The highest recorded tissue drug levels were in the lymph nodes (4–6 μM), followed by kidney and liver (2.5, 2.0 uM, respectively).

**Conclusions:**

We obtained critical information regarding the PK and toxicity of MnBuOE in dogs. The acute drug reaction and tachycardia post-injection have not been described in other species and may be specific to canines. The high tissue drug levels in lymph nodes have not been previously reported. MnBuOE accumulation in lymph nodes has important implications for the utility of adjuvant MnBuOE to treat lymphoma. With MnBuOE lymph node accumulation, reduction in the dose and/or administration frequency could be possible, leading to reduced toxicity.

**Electronic supplementary material:**

The online version of this article (doi:10.1007/s00280-017-3372-z) contains supplementary material, which is available to authorized users.

## Introduction

**Fig. 1:**
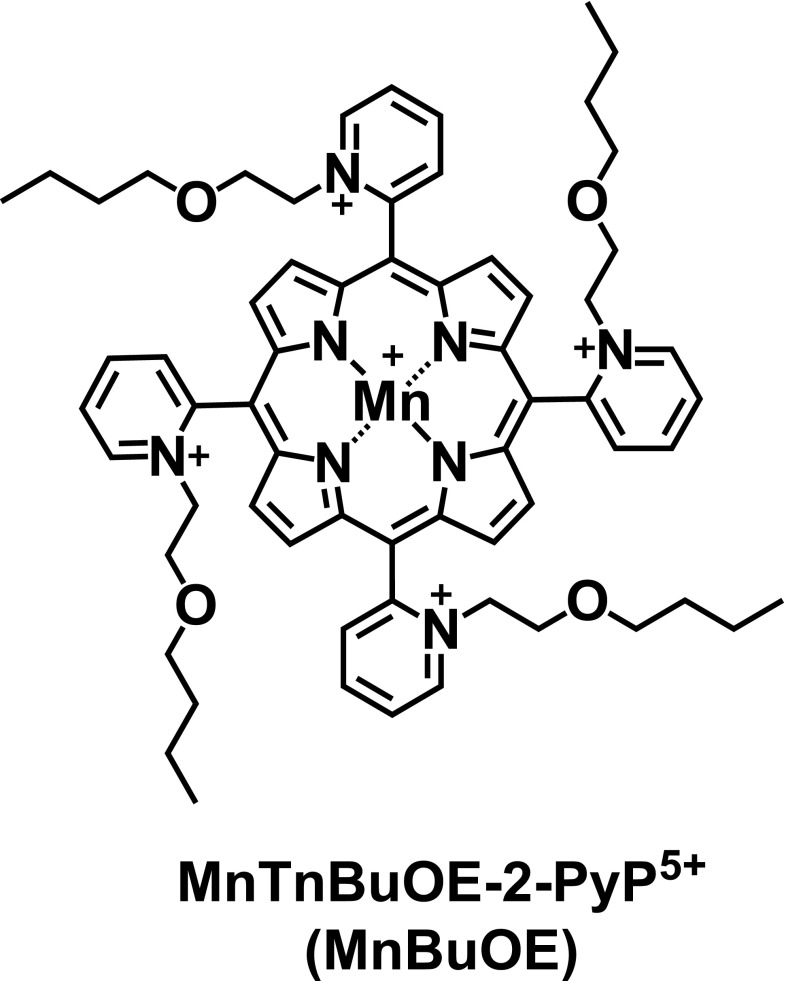
Chemical structure of the studied compound

Cancer therapy should operate with a wide therapeutic index whereby tumor cells are preferentially killed and normal tissues are protected. Unfortunately, the most common cancer treatments are toxic to both tumor and normal tissues and the therapeutic index is narrow. Many cancer therapeutics increase cellular oxidative stress and this stress can induce cell death. Increased oxidative stress has been observed after radiation therapy, several common chemotherapy agents, and even targeted agents [1–4]. While the increase in oxidative stress contributes to tumor cell cytotoxicity, it can also be damaging to normal tissues. We have designed a novel manganese porphyrin, MnTBuOE-2-PyP^5+^ (MnBuOE) (Fig. 1). MnBuOE is a superoxide dismutase (SOD) mimic, which has been shown to sensitize tumor cells to chemotherapy and radiotherapy while protecting normal tissue by modulating tissue reduction-oxidation (redox) status [5–9]. Recently, we have reviewed the structure–activity relationships and impact of these manganese porphyrin therapeutics on cellular redox-based signaling pathways [10].

MnBuOE has divergent effects on tumor and normal tissue due to the inherent differences in basal cellular oxidative stress levels. Normal tissues have low levels of reactive species, and, in this context, MnBuOE serves as an antioxidant, marginally suppressing NF-kB activity, triggering anti-inflammatory responses, preventing cycling oxidative stress, and protecting normal cells from damage [10]. In tumors, however, an excessive baseline level of oxidative stress exists. The oxidative stress is driven by accumulation of the long-lasting reactive oxygen species, hydrogen peroxide. This excessive baseline level of oxidative stress is further enhanced by cancer therapies. As a cancer therapy adjuvant, MnBuOE serves as a pro-oxidant, stimulating the peroxide-induced oxidation of NF-kB, suppressing mitochondrial respiration, inhibiting glycolysis, and driving tumor cytotoxicity [10].

MnBuOE is primed for clinical use as an adjuvant cancer therapy and normal tissue protector. The compound has been optimized for pharmacodynamics with minimal toxicity and has entered Phase I/II Clinical Trials at Duke University as a radioprotector of normal tissue for patients with glioma who are treated with radiotherapy and temozolomide (Clinical Trial NCT02655601, clinicaltrials.gov).

A manganese porphyrin derivative from MnBuOE, MnTE-2-PyP5+, has shown benefits as a chemosensitizer for lymphoma. Lymphoma is a hematologic malignancy which develops within the lymph nodes. Jaramillo et al. demonstrated that MnTE-2-PyP5+ enhanced the chemotherapeutic response of murine lymphoma cell lines and primary human lymphoma samples *in vitro* and *ex vivo*, respectively [11, 12]. Chemosensitization of lymphoma cells by adjuvant manganese porphyrin would have significant implications for patients undergoing chemotherapy. As with many preclinical models, rodent models of lymphoma may not accurately portray the natural biology of the disease [13, 14]. Instead, important information regarding the clinical use of adjuvant MnBuOE in treating lymphoma may be gleaned from a comparative canine oncology trial [15].

Dogs develop a broad spectrum of naturally occurring cancers that share strong similarities with human cancers: they are an out-bred population, and, like human patients, pets receive state-of-the-art medical care; studying dogs with cancer offers a remarkable opportunity for preclinical modeling [15, 16]. For the interest of this study, we are considering canine lymphoma and the potential to perform a comparative oncology clinical trial to investigate the utility of MnBuOE as an adjuvant to canine lymphoma therapy. Positive results from a canine lymphoma trial would provide support for moving MnBuOE into clinical trials as an adjuvant to human lymphoma therapy. Many similarities exist between human and canine lymphoma: both are spontaneously occurring and genetically diverse, most patients are diagnosed with the Non-Hodgkin’s type of lymphoma, and they are both treated with CHOP-based chemotherapy treatment protocols. The clinical value in studying pet dogs with lymphoma as a model of the human disease is actively being researched and reported [17, 18].

Before introducing MnBuOE into the veterinary clinic to perform a companion canine lymphoma clinical trial, the goal of this study was to determine the safety and optimal dosing regimen of MnBuOE in normal dogs.

## Materials and methods

### Dogs

All animal care and experimental procedures were approved by and complied with the regulations of the North Carolina State University Institutional Animal Care and Use Committee, as described in the Guide for Care and Use of Laboratory Animals published by the National Institutes of Health (NIH Publication No. 85-23, revised 1985). Three adult male Beagle dogs (4 years old; one reproductively intact, two neutered; weight: 11.6–13.1 kg) were used for all phases of this study. Baseline physical examination and systemic health analysis with standard laboratory assessments (complete blood cell count, biochemistry panel, urinalysis) confirmed that the dogs were in good health prior to initiation of the study.

### MnBuOE structure and preparation

The chemical structure of MnBuOE is shown in Fig. 1. MnBuOE was synthesized and purified according to the procedures described elsewhere [19, 20].

### Monitoring

Safety was assessed by evaluation of standard physical and laboratory parameters of systemic health. A full physical examination was performed prior to initiation of the study and on each treatment and sampling day. Indirect blood pressure monitoring was performed using Cardell Vet Monitor 9401 Blood Pressure Monitor. Pulse was quantified as heart beats per minute according to manual counts, as well as quantified with a blood pressure monitor. There was good correlation between pulse rates counted with manual methods compared to the blood pressure monitor (Figure S1). Standard laboratory assessments (complete blood cell count, biochemistry panel, urinalysis) were repeated throughout the phases of the study to assess systemic health.

### Initial dose for single-dose pharmacokinetic and MTD studies

Prior toxicokinetic studies in mice and nonhuman primates in which MnBuOE was administered subcutaneously every 3 days for 5 weeks demonstrated no adverse effects level (NOAEL) to be 12 mg/kg loading dose and 2 mg/kg maintenance dose in mice and 6 mg/kg loading dose and 2 mg/kg maintenance dose in nonhuman primates [21]. Pharmacodynamic studies in mice demonstrated that a dose of 1 mg/kg is adequate to achieve pharmacologically active levels in normal and tumor tissues [5, 6, 10, 22, 23]. Thus, 1 mg/kg was selected as a safe initial dose for the MTD and single-dose PK studies in this work.

### Single-dose pharmacokinetic study

Intravenous catheters were placed in the jugular vein of each dog under heavy sedation (dexmedetomidine, 500 mcg/m^2^) 24-h prior to initiation of the pharmacokinetic study to facilitate blood sampling. Baseline pulse and blood pressure (Cardell Vet Monitor 9401 Blood Pressure Monitor) were recorded prior administration of MnBuOE. MnBuOE (5.64 mg/mL) was injected subcutaneously in the dorsal intrascapular region. Following injection, 3–6 mL of blood was collected after 10 min, 30 min, 1 h, 2 h, 4 h, 6 h, 8 h, 24 h, 48 h, 72 h, 7 days, and 14 days into lithium heparin tubes (Vacutainer, 1.3 mL). Whole blood was centrifuged at 1500×*g* for 20 min, and plasma was collected and stored in 1% citric acid at −80 °C.

### Defining maximally tolerated dose

The maximally tolerated dose (MTD) to be used as an initial (loading) dose for the multiple dose pharmacokinetic study was defined as the dose of MnBuOE which did not induce clinically intolerable side effects in the dogs.

### Multiple dose pharmacokinetic study

The dosing regimen selected for the multiple dose pharmacokinetic study was based on the *t*_1/2_ defined in the single dose pharmacokinetic study. Blood samples were collected prior to and 1 h following administration of MnBuOE. Blood samples were processed and stored as described for the single-dose pharmacokinetic study. Pulse was counted manually prior to and 1 h following administration of MnBuOE. This multiple dose pharmacokinetic study was performed twice to ensure plasma drug levels were maintained as predicted and that these results were reproducible. The first study involved a treatment period of 3 weeks, and the second study spanned a treatment period of 2 weeks leading up to euthanasia with necropsy and tissue harvest.

Samples from organs and tissues of interest were harvested during necropsy and placed over dry ice until stored at −80 °C. Drug concentration was analyzed by LC/MS-MS.

### Histopathologic evaluation

Tissue samples collected at necropsy were stored in 10% neutral buffered formalin, paraffin embedded, sectioned, and stained with hematoxylin and eosin. Histopathologic evaluations of the tissues were performed by a veterinary pathologist (KGB).

### Measurement of MnBuOE in plasma and organs

#### Plasma and tissue processing

Organs were cryo-pulverized in a Bessman tissue pulverizer (BioSpec Products, Bartlesville, OK) under liquid nitrogen and then homogenized in a rotary homogenizer (PTFE pestle and glass tube) with two volumes of deionized water. An aliquot of either plasma or tissue homogenate was transferred into a 2-mL polypropylene screw-cap vial and a double volume of 1% HCl in methanol was added, agitated in FastPrep apparatus (Q-biogene, Carlsbad, CA) at speed 6.5 for 20 s, and centrifuged 10 min at 16,000*g*. For plasma an aliquot of the supernatant was pipetted into a HPLC autosampler polypropylene vial and 80 μL of 0.1% heptafluorobutyric acid (HFBA) in water was added. It was followed by another cycle of centrifugation for 5 min at 4500*g* (4 °C), after which the sample was immediately analyzed by LC-MS/MS. For other organs, an aliquot of the supernatant was pipetted into a 5-mL polypropylene tube (10 × 50 mm) and the solvent was completely removed in a Savant Speed-Vac evaporator at 40 °C within 1 h. The dry residue was dissolved in 20  μL of mobile phase B (see below) and sonicated for 5 min; then 80 μL of mobile phase B was added, the mixture was sonicated again for 5 min, and centrifuged for 5 min at 4500*g* at 4 °C. Finally, the tube content was transferred to the HPLC autosampler polypropylene vial equipped with silicone/polytetrafluoroethylene septum, followed by another cycle of centrifugation for 5 min at 4500*g* (4 °C), placed in autosampler 4 °C, and analyzed by LC-MS/MS.

#### Liquid chromatography-tandem mass spectroscopy (LC-MS/MS)

Quantitative analysis of MnBuOE was performed on a Shimadzu 20A series HPLC (LC)—Applied Biosystems MDS Sciex 5500 QTrap tandem mass spectrometer (MS/MS) at PK/PD Core Laboratory of Duke Cancer Institute. The use of HFBA as an ion-pairing agent increases overall lipophilicity/volatility and greatly improves retention and ionization efficiency of the analytes, affording an abundance of [MnP^5+^ + 2HFBA^−^]^3+^ and [MnP^5+^ + 3HFBA^−^]^2+^ ions. Solvents employed were: *A* = 9:1 water:acetonitrile (0.05% HFBA); *B* = acetonitrile (0.05% HFBA). Analytical column: 2 × Phenomenex AJ0-4287, C18, 4 × 3 mm at room temperature. Elution gradient: 0–1 min 0–70%B, 1–2 min 70%B, 2–2.1 min 70–100%B, 2.1–2.6 min 100%B, 2.6–2.7 min 100–0%B. Run time: 4 min. Mass transitions used for quantification: MnTnBuOE-2-PyP^5+^ at *m*/*z* = 857.3/599 and MnTnBuOE-2-PyP^5+^-d_8_(internal standard) at *m*/*z* = 862.2/603.9. Calibration samples in 1–1000 nM or 0.1–10 μM range (depending on the expected levels) were prepared by adding known amounts of serially diluted pure standards into plasma or corresponding tissue homogenates and were analyzed along with study samples. Response was calculated as the ratio between the standard peak area and internal standard peak area.

## Results

### Single-dose pharmacokinetic study

**Fig. 2:**
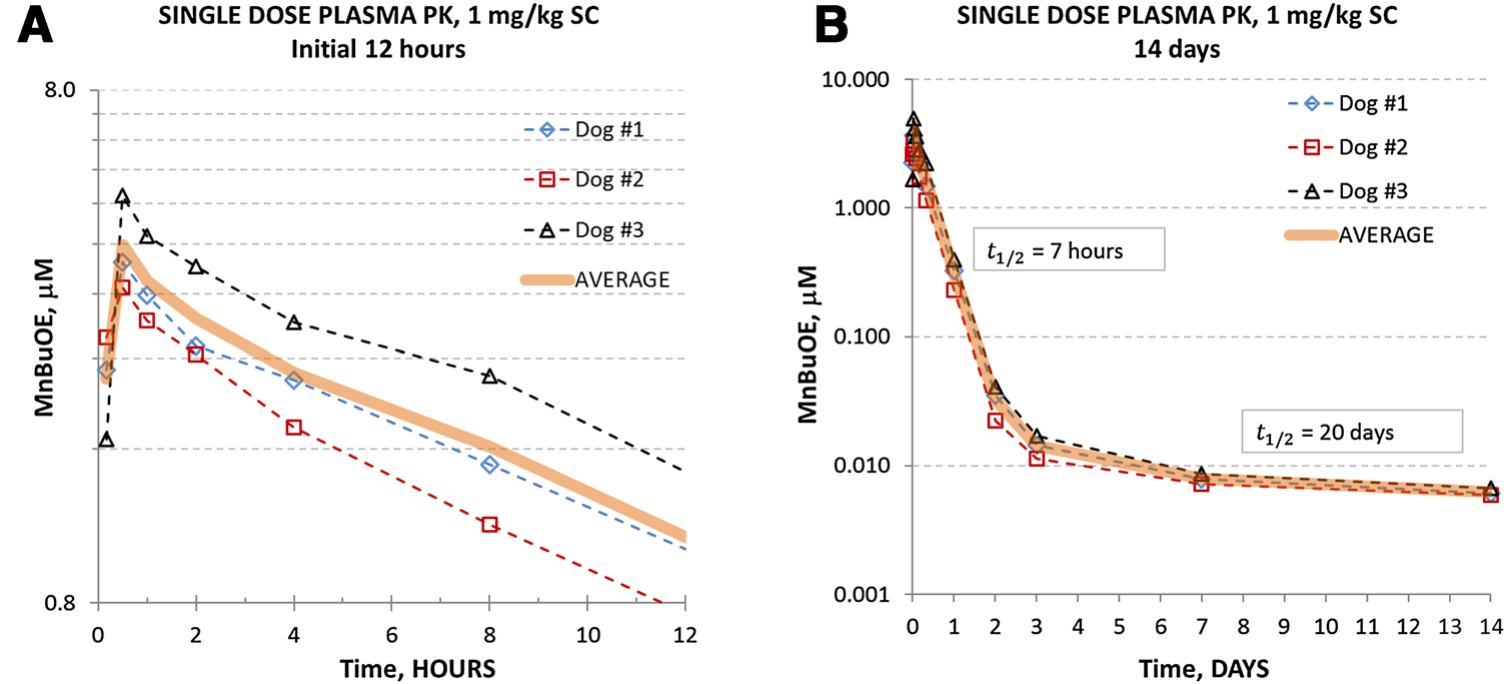
Pharmacokinetic profile of MnBuOE in dog after single subcutaneous injection. **a** Concentration of MnBuOE in plasma during initial 12 h after single subcutaneous injection of 1 mg/kg (in 100 μL saline) to 3 dogs (*average line* presented for vizualization only). Absorption from subcutaneous space (*T*_max_ = 30 min) is followed by a complex distribution into various organs/compartments. **b** A single (linear in log-lin plot) plasma elimination process, leaving only 1% drug remaining, starts after first 8 h and ends in 2 days, followed by a complex elimination profile, presumably from cellular compartments of various affinity for the drug, extending beyond 2 weeks

The complex PK profile obtained is presented in Fig. 2a, b. Relevant PK parameters (e.g. *C*_max_, *T*_max_, AUC, clearance, *t*_1/2_) were obtained by compartmental and non-compartmental modules within WinNonlin v. 2.1 software (Pharsight Corp. Cary NC) and are presented in Table 1. Plasma MnBuOE levels peaked at 30 min post-injection with a mean concentration of 3.993 μM (Fig. 2a), followed by a complex tissue distribution profile (non-linear trace in log-linear plot) within first 6 h post injection (Fig. 2a). From 6 to 48 h, however, a single exponential (linear in log-linear plot, Fig. 2b) elimination process from the central compartment (e.g. plasma and weakly bound drug to plasma proteins, membranes, etc) was observed with the half-life *t*_1/2_ = 7 h, leaving only 1% of MnBuOE remaining in plasma. From 48 h to 14 days (Fig. 2b), a complex process (presumably a slow elimination from “deep” compartments) was observed with the estimated “terminal” half-life of 20 days. Selected PK parameters from compartmental and non-compartmental PK calculations are presented in Table 1. Compartmental (first-order absorption—2 compartment model) calculations including 0–48 h data were performed on the averaged PK profile for the purpose of obtaining PK parameters needed for the simulation of the multi-dose regimen.

**Table 1:** PK parameters

Win nonlin PK calculation	Non-compartmental		First order absorption two compartments	
PK parameter	Value	Std. error	Value	Std. error
*t*_max_ (h)	0.5	0	0.46	0.46
*C*_max_ (mg/mL)	5.00	0.64	4.83	0.21
AUC_0–48h_	52.09	7.89	N/A	N/A
AUC_infinite_	57.82	7.65	42.52	19.24
*t*_1/2_ (4–48 h) (h)	7.13	0.13	6.64	4.24
*t*_1/2_ (7–14 day) (day)	21.24	2.11	N/A	N/A
V/F (4–48 h process) (L/kg body weight)	0.21	0.03	0.14	0.76
V/F (7–14 day process) (L/kg body weight)	13.42	2.92	N/A	N/A
CL/F (L/h/kg body weight)	0.020	0.003	0.02	0.01

Non-compartmental parameter estimation module within WinNonlin software was performed on concentration/time sets from individual dogs after single 1 mg/kg dose to obtain critical PK parameters. Compartmental modeling (absorption +2 compartments) using average single-dose profile was performed to estimate the PK parameters in order to simulate the multi-dose regimen. Since the 4–48 h process dominates, selected PK parameters are in very good agreement; complexity of the overall profile and scarcity of the early data preclude rigorous compartmental modeling of all the processes observed

### Defining maximally tolerated dose

#### Results at 1 mg/kg

**Fig. 3:**
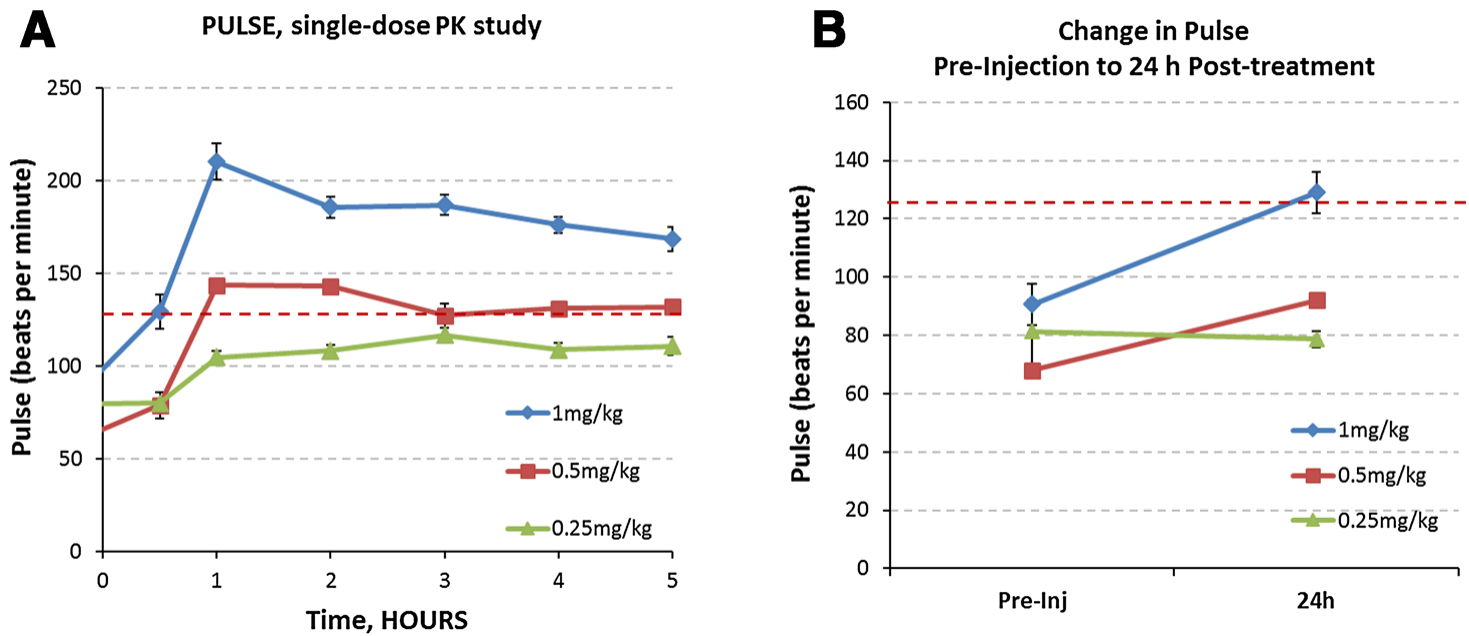
Pulse measured after single MnBuOE injection. **a** Subcutaneous administration of MnBuOE induced a dose-responsive acute and persistent increase pulse rate (*n* = 3/3). A severe tachycardia was documented following administration with 1 mg/kg MnBuOE. Reducing the MnBuOE dose to 0.5 mg/kg resulted in a mild to moderate tachycardia. Reducing the dose to 0.25 mg/kg MnBuOE increased the pulse rate but did not induce a clinical tachycardia. *Dotted line* indicates the defined acceptable resting pulse rate for the dogs. **b** Subcutaneous administration of MnBuOE induced a dose-responsive increase in pulse rate that persisted 24 h post-injection. Treatment with 0.25 mg/kg MnBuOE did not alter pulse rate 24 h post-injection

Administration of the 1 mg/kg dose resulted in unexpected and unacceptable toxicities in all of the dogs. The first toxicity was an acute anaphylactic drug reaction which developed 15–20 min following injection of MnBuOE. This reaction was characterized by hyperemic mucus membranes, head shaking, urticaria, restlessness, and protrusion of the third eyelids. These clinical signs were resolved with treatment with an antihistamine medication (diphenhydramine, 2 mg/kg). The second toxicity was a severe sinus tachycardia, which peaked 1 h after MnBuOE injection. Average heart rates at this time point were 210 beats per minute (Fig. 3a). By comparison, resting heart rate for these dogs averaged 88 beats per minute. This tachycardia persisted for at least 6 h (Fig. 3a), and pulses remained increased compared to resting heart rates 24 h later (Fig. 3b). The tachycardia was not associated with changes in blood pressure (Figure S2), nor was the tachycardia alleviated by the antihistamine medication (diphenhydramine) or intravenous fluid therapy (Figure S3).

Local drug reactions in the subcutaneous site of injection occurred with two of the dogs. One dog developed non-painful, mild hyperemia the time of injection, and this resolved without treatment 48 h later. The other dog developed a non-painful, subcutaneous thickening 3 days after MnBuOE injection; this reaction resolved without treatment 4 days later. Alkaline urine (pH = 9) was documented in two of the three dogs at 48 h after MnBuOE administration (Table 2).

**Table 2:** Health assessments

Laboratory test	Pre-treatment	48 h post 1 mg/kg	48 h post 0.25 mg/kg	3 weeks post 0.25 mg/kg
Laboratory health assessments				
Complete blood cell count	Unremarkable	Unremarkable	Unremarkable	Stress leukogram (exogenous glucocorticoids) (*n* = 3/3)
Chemistry panel	Unremarkable	Unremarkable	Unremarkable	Mild hypocholesterolemia (*n* = 3/3) Mildly increased liver enzyme activity (ALP, ALT) (*n* = 3/3) Slight to mild metabolic acidosis (increased anion gap) (*n* = 3/3)
Urinalysis	Unremarkable	Alkaline urine (pH = 9) (*n* = 2/3)	Alkaline urine (pH = 9) (*n* = 1/1)	Alkaline urine (pH = 9) (*n* = 2/3)

Affected organ/tissue	Diagnosis	Incidence	Characteristics	Incidence
Histopathologic evaluation				
Lung	Bronchointerstitial/interstitial pneumonia	*n* = 3/3	Multifocal, mild, neutrophilic, sterile Multifocal, moderate, neutrophilic sterile	*n* = 2/3 *n* = 1/3
Liver	Hydropic degeneration	*n* = 3/3	Marked, multifocal to diffuse, random Marked, multifocal to diffuse, centrilobular to random Marked, diffuse, panlobular, random	*n* = 1/3 *n* = 1/3 *n* = 1/3
Kidney	Tubular degeneration and necrosis	*n* = 3/3	Acute, multifocal, mild Acute multifocal, moderate	*n* = 2/3 *n* = 1/3
Subcutaneous adipose	Necrosuppurative/granulomatous steatitis	*n* = 3/3	Multifocal, mild	*n* = 3/3

MnBuOE at 0.25 mg/kg and concurrent oral medications. Slight to mild metabolic acidosis (*n* = 3/3) was also seen following this multiple dosing protocol. Single doses of MnBuOE (1, 0.25 mg/kg) and multiple doses of MnBuOE at 0.25 mg/kg did not alter blood cell volumes. Three weeks of treatment with prednisone induced shifts in white blood cell counts in a pattern consistent with administration of exogenous glucocorticoids. Tissue samples were stored in 10% neutral buffered formalin, paraffin embedded, sectioned, and stained with hematoxylin and eosin. Histopathologic evaluations of the tissues were performed by a veterinary pathologist (KB). Varying degrees of bronchointerstitial/interstitial pneumonia (*n* = 3/3), hydropic degeneration of the liver (*n* = 3/3), tubular degeneration and necrosis of the kidney (*n* = 3/3), and necrosuppurative/granulomatous steatitis in the subcutaneous adipose tissue of the injection site (*n* = 3/3) were identified

#### Results at 0.5 mg/kg

The dose of MnBuOE was reduced to 0.5 mg/kg and tested in one dog. MnBuOE was injected 1 h after pre-administration of diphenhydramine (2 mg/kg), famotidine (0.5 mg/kg), and prednisone (1 mg/kg). The anaphylactic reaction seen with MnBuOE alone did not occur with pretreatment with these drugs. Based on this observation, the remainder of the studies reported in this paper included pre-medication with anti-histamines and steroids. No local drug reaction at the injection site was noted. However, the dog still developed a moderate tachycardia following administration of the 0.5 mg/kg dose, peaking at 1 h after injection with an average of 144 beats per minute; the heart rate 24 h later remained slightly increased (Fig. 3a, b). This degree of tachycardia was deemed too high to justify using this drug at this dose in a companion canine lymphoma clinical trial.

#### Results at 0.25 mg/kg

No anaphylactic or local drug reactions were observed in the first dog treated with 0.25 mg/kg, so this dose was repeated in the other two dogs. A mild increase in heart rate developed (range 104–116 beats per minute) following injection, but had normalized by 24 h (Fig. 3a, b). This level of increase in heart rate was considered clinically acceptable for a canine lymphoma trial. Laboratory health assessments were repeated 48 h following administration of 0.25 mg/kg MnBuOE. No remarkable changes in serum biochemistries were seen. Alkaline urine (pH = 9) was seen in the one dog from which urine was analyzed (Table 2). Based on these findings, 0.25 mg/kg of MnBuOE was defined as the MTD.

### Multiple dose pharmacokinetic studies

**Fig. 4:**
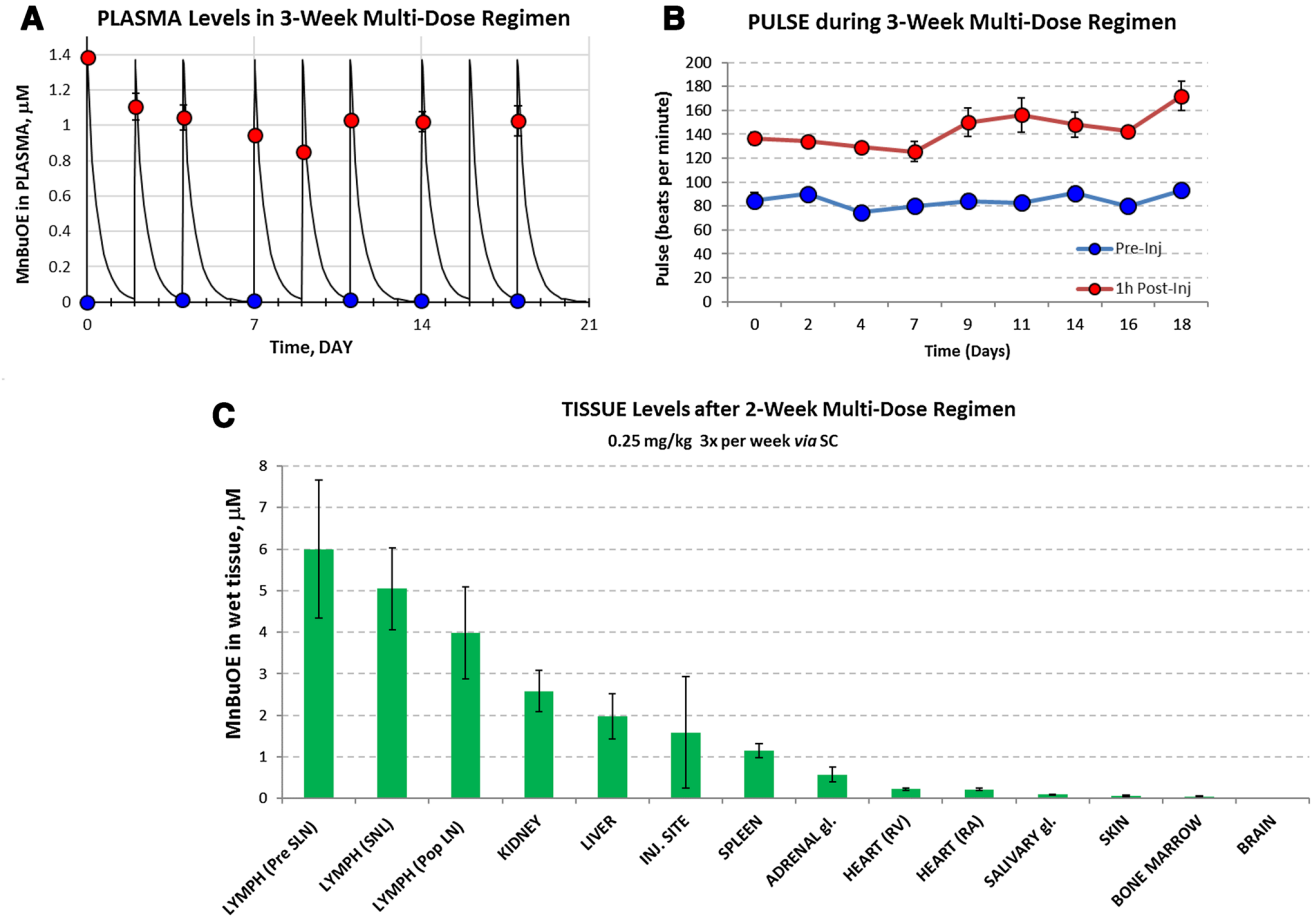
Tissues after 2-week multi-dose subcutaneous treatment. **a** MnBuOE was administered subcutaneously at 0.25 mg/kg every Monday, Wednesday, and Friday for 3 weeks. Plasma MnBuOE was measured pre-injection and 1 h post-injection. MnBuOE concentration was highest following the initial dose (1.4 μM) then plateaued with subsequent doses with a range of approximately 0.85 to 1.1 μM. Owing to 7 h half-life of the elimination and even shorter initial distribution-phase half-life, MnBuOE was essentially cleared prior to each subsequent dose; consequently, no plasma accumulation was observed. **b** Subcutaneous administration of MnBuOE at 0.25 mg/kg increased pulse rate 1 h post-injection. When MnBuOE was administered Mon/Wed/Fri for 3 weeks, the change in pulse increased over time for both treatment week and day of the week. Pre-injection pulse rate was not altered with multiple dosing of MnBuOE. **c** Dogs received 0.25 mg/kg MnBuOE Mon/Wed/Fri for a total of five doses prior to euthanasia. Tissues were harvested 48 h post-last injection. Even after 48 h, most tissues retained higher concentration of the drug than the *C*_max_ in plasma, suggesting that the drug accumulates during the given dosing regimen. Drug levels were highest in peripheral lymph nodes (prescapular, submandibular, popliteal) and lowest in brain tissue

MnBuOE was administered at 0.25 mg/kg Monday, Wednesday, and Friday (MWF). Dogs were treated concurrently with diphenhydramine (2 mg/kg every 12 h), famotidine (0.5 mg/kg every 12 h), and prednisone (1 mg/kg every 12 h) throughout these studies. Blood/plasma was collected at two time points: 1 h post dose (expected high levels but “safely” away the peak at *C*_max_ = 0.5 h) and just prior to injection (trough level). The results are presented on Fig. 4a. As expected, the trough levels measured were very low. An important finding is that no accumulation of the drug in plasma was observed during the entire long-term treatment, i.e. high levels never exceeded the value observed after the first injection (based on which the MTD-0.25 mg/kg was established). This would imply that no change in the expected mild acute side effects should be observed. Mildly to moderately increased heart rate 1 h after each injection was observed (Fig. 4b). The change in pulse increased over time for both treatment week and day of the week. Baseline pulse prior to injection was not altered with multiple doses of MnBuOE.

#### First study

Laboratory health assessments were repeated on the final day of the initial 3-week study. Mildly increased liver enzyme activity (mean ALP 203 IU/L, reference range 16–140 IU/L; mean ALT 133 IU/L, reference range 12–54 IU/L) and mild hypocholesterolemia (mean 96 mg/dL, reference range 124–344 mg/dL) were documented in all three dogs, as well as a slight-to-mild anion gap metabolic acidosis (mean 21.5, reference range 11.2–19.9) (Table 2). Alkaline urine (pH = 9) was reported for two of the three dogs. Prolonged treatment with prednisone induced shifts in white blood cell counts in a pattern consistent with administration of exogenous glucocorticoids (*n* = 3/3). One of the dogs developed a urinary tract infection and was successfully treated with enrofloxacin (10 mg/kg every 24 h for 10 days).

#### Second study

Following a 44-day wash-out period, the dogs were treated with the MWF protocol, beginning on a Monday, for a total of five doses of MnBuOE. Forty-eight hours after the last dose of MnBuOE, the dogs were euthanized (intravenous injections of acepromazine (0.2 mg/kg), butorphanol (2.0 mg/kg), and pentobarbital sodium (390 mg/mL; 1 mL/lb) and tissues were harvested.

### Histopathologic evaluation

Varying degrees of bronchointerstitial/interstitial pneumonia (*n* = 3/3), hydropic degeneration of the liver (*n* = 3/3), tubular degeneration and necrosis of the kidney (*n* = 3/3), and necrosuppurative/granulomatous steatitis in the subcutaneous adipose tissue of the injection site (*n* = 3/3) were identified (Table 2). Lymph nodes appeared mildly reactive, but this was not considered clinically significant.

### Tissue drug levels

The highest recorded tissue drug levels were in the lymph nodes (3.98–5.99 μM), followed by the kidney and liver (2.58, 1.97 μM, respectively) (Fig. 4c).

## Discussion

In this study, we obtained critical information regarding the PK and toxicity of MnBuOE in normal dogs as a prelude to a planned clinical trial in canine patients with lymphoma.

The *t*_1/2_ of MnBuOE was defined as 7 h via primary elimination and 20 days via secondary processes. Following a multi-dose PK study, the highest recorded tissue drug levels were in the peripheral lymph nodes (3.98–5.99μM), followed by the kidney and liver (2.58, 1.97 μM, respectively).

The most important result from the single-dose 14-day PK study was that the 99% of drug was eliminated from the plasma after 48 h. This implies that, in a multi-dose treatment protocol, the same initial dose may be given every 2 days without the danger of drug accumulation above the established single-dose MTD. The data obtained from 48 h to 14 days suggest a slow elimination from multiple “deep” compartments (cell cytosol and organelles). Another important result is that *C*_max_ (4 μM) obtained in this study with dogs is much higher than the value observed in mice [*C*_max_ ~0.3 μM (dose adjusted from 6 mg/kg [21]) and *C*_max_ = 1 μM (1 mg/kg, unpublished data)], rats [*C*_max_ = 1 μM (1 mg/kg, unpublished data)], and non-human primates [*C*_max_ = 1.6 μM (1 mg/kg [21])]. This can explain the unexpected serious side effects and the lower than expected MTD established in this study with dogs.

Based on the results from the multiple-dose PK study, it was confirmed that the plasma peak concentration is controlled (no accumulation observed over time, Fig. 4a). This suggests that the acute toxicity signs should not worsen over the course of long-term therapy. Tissue analysis revealed accumulation of the drug within organs. Particularly encouraging for this study is that the highest tissue drug levels were observed in lymph nodes. If plasma peak concentration were the only controlling factor for the acute toxicity, the observed tissue accumulation would be only beneficial for the treatment. However, pulse data as well as laboratory assessments suggest that controlling plasma level is not sufficient and that long-term multiple-dose MTD should be lower than 0.25 mg/kg and/or frequency of dosing extended to once-weekly, depending on the application.

As we prepare to move toward testing the utility of adjuvant MnBuOE in the treatment of naturally occurring canine lymphoma, the finding that the highest drug levels were measured in the lymph nodes is particularly encouraging. The high tissue drug levels in lymph nodes have not been documented previously; this was the first animal study to perform such measurements. Although it is a different cell type, we have reported a threefold higher accumulation of manganese porphryins in the nucleus of macrophages compared to the cytosol [24]. It is likely that MnBuOE is also accumulating in the nucleus of lymphocytes. Lymphocytes are a cell type with a very high nuclear to cytosolic ratio and they are tightly packed with a high cell density within the parenchyma of lymph nodes [25]. The preferential accumulation of MnBuOE in lymph nodes is likely due to both the accumulation of the drug within the nucleus of lymphocytes and the high lymphocyte cell density in the lymph node. Although MnBuOE lymph node accumulation was recorded in normal, healthy dogs in this study, it is important to consider that manganese porphyrin compounds also accumulate in tumor tissue. Recently, we demonstrated that MnTE-2-PyP5+, a manganese porphyrin compound similar to MnBuOE, accumulates preferentially in tumor tissue compared to normal tissue [22]. Evidence that manganese porphyrin compounds accumulate in both primary tumor tissue and lymph nodes strengthens the justification to use MnBuOE in a canine lymphoma trial. For these reasons, patients with lymphoma may benefit substantially from adding adjuvant manganese porphyrin to chemotherapy protocols given its chemosensitization properties against lymphoma cells [11, 12]. The high lymph node drug level is also an important finding when considering other types of cancers which metastasize to lymph nodes; for these cancers, improved treatment outcomes may also arise from adjuvant MnBuOE.

The greatest obstacle in treating both human and canine lymphoma is the development of drug resistance. Treatment with adjuvant MnBuOE may be a way around this drug resistance. The combination of manganese porphryins and certain chemotherapy agents creates an environment of high oxidative stress within tumor cells. This pro-oxidant mechanism of chemosensitization by manganese porphyrins creates a scenario whereby lymphoma cells will be less likely to survive and develop drug resistance [11, 12]. This could lead to lengthened remission duration. Yet another advantage to performing comparative oncology studies in dogs is the ability to initiate a Phase I clinical trial prior to patients receiving and failing standard-of-care treatment protocols due to drug resistance. Given that phase I human lymphoma clinical trials typically are only able to evaluate treatment outcomes for patients who have already failed frontline therapy, reports of improved clinical outcomes for dogs treated upfront with experimental therapies, such as adjuvant MnBuOE, could provide support for moving forward with subsequent human clinical trials.

Although there is more flexibility in performing a comparative oncology trial, it is imperative that the canine patients are treated safely and responsibly; after all, these dogs are companion animals. Therefore, before testing MnBuOE in canine cancer patients, it was necessary to understand the safety and optimal dosing regimen in normal dogs. Prior studies in mice demonstrated pharmacological effects in tumor and normal tissues with 1 mg/kg subcutaneous administration of MnBuOE [5, 6, 22]. This dose is well below the MTD established for mice and non-human primates [21, 23]. Thus, 1 mg/kg was selected as a safe initial dose for MTD and single-dose PK studies in dogs which would also provide accurate measurement of MnBuOE in plasma even 14 days after injection. However, the 1 mg/kg dose of MnBuOE induced an anaphylactic drug reaction and a severe, prolonged tachycardia in the dogs. The acute drug reaction was prevented with premedications (steroids, anti-histamines) and the tachycardia was alleviated by reducing the MnBuOE dose. Neither intravenous fluid therapy nor anti-histamine medication affected the tachycardia, indicating that this toxicity is most likely a primary tachycardia and not secondary to hypotension. Consequently, the MTD was lowered to 0.25 mg/kg. Aside from a mild to moderately increased heart rate 1 h post-injection that increased in severity over time, the dogs had no clinical evidence of toxicity throughout the multi-dose PK study at this dose. This change in heart rate throughout the multi-dose PK study is most likely due to accumulation of MnBuOE in the cardiac tissue. The acute anaphylactic drug reaction and tachycardia post-injection have not been described in other species and may be specific to canines.

Laboratory tests performed throughout the studies identified changes to the organ systems functions of the dogs when treated with MnBuOE. Following treatment of MnBuOE as single doses of 1 and 0.25 mg/kg, as well as prolonged treatment in the first multi-dose study, alkaline urine was documented (*n* = 1–2/3). A slight-to-mild high anion gap metabolic acidosis developed in all three dogs following the 3-week multi-dose PK study. These results combined are indicative of renal damage. One dog developed a urinary tract infection during the study; however, this was most likely secondary to introduction of bacteria into the bladder during urine collection via cystocentesis. To prevent the anaphylactic drug reaction associated with MnBuOE administration, dogs were treated with prednisone and antihistamine medications throughout the multi-dose PK study. Prednisone can induce changes in liver enzyme activity, and, in fact, mildly increased liver enzyme levels were recorded in the three dogs; however, mild hypocholesterolemia was also found in the three dogs, which may indicate that the changes in the liver are due to damage from the MnBuOE.

Histopathologic evaluation of tissues revealed mild to moderate inflammatory and degenerative changes in the kidney, liver, and lungs. Consistent with the laboratory results, acute, mild to moderate tubular degeneration and necrosis of the kidney was reported (*n* = 3/3), as well as marked hydropic degeneration of the liver (*n* = 3/3). Interestingly, mild to moderate sterile bronchointerstitial/interstitial pneumonia was discovered (*n* = 3/3). While MnBuOE causing these changes in the lungs cannot be ruled out, the characteristics of this pneumonia could also point to an acute injury from inhalation of a noxious cleaning agent from the kennel in which they were housed. Finally, mild necrosupporative/granulomatous inflammation was found in the subcutaneous adipose tissue of the injection site (*n* = 3/3). These findings have not been reported in other species and may be specific to canines.

Again, because manganese porphyrin compounds accumulate in primary tumors and lymph nodes, it may be possible to reduce the treatment dose and/or frequency of administration of MnBuOE for a canine lymphoma trial while maintaining clinical efficacy in order to reduce or prevent the identified toxicities.

## Electronic supplementary material

Below is the link to the electronic supplementary material.

**Supplemental Fig. 1** Correlation of pulse measured by machine or manual counts. Pulse was quantified via machine (indirect blood pressure monitor) and manual counting (palpation or auscultation). There was a strong correlation (0.8973) between pulse quantified with the two methods. Pulse was quantified via manual counting for the multiple dosing experiment. **Supplemental Fig. 2** Variations in indirect blood pressure following administration of single dose of MnBuOE. Subcutaneous administration of MnBuOE caused subclinical fluctuations in recorded indirect blood pressures. Administration of MnBuOE at 0.25mg/kg resulted in the most stable maintenance of initial blood pressures. **Supplemental Fig. 3** Variations in heart rate and blood pressure following administration of single dose of MnBuOE. Dog 2 and Dog 3 were treated with diphenhydramine (2 mg/kg intramuscularly) upon the first evidence of anaphylactic drug reaction (20 minutes post-injection MnBuOE). Dog 3 was treated with 3 intravenous boluses of lactated ringer’s solution (10–12 mL/kg over 15 minutes). Tachycardia persisted following treatment with diphenhydramine (n = 2/2) and intravenous fluid therapy (n= 1/1). The effects of treatment with diphenhydramine and/or intravenous fluid therapy on indirect blood pressure measurements are unclear (PDF 165 kb)
