# Supplementary material for: Potential for a novel manganese porphyrin compound as adjuvant canine lymphoma therapy
Source: Cancer Chemother Pharmacol. 2017 Jul 6;80(2):421–31. doi: 10.1007/s00280-017-3372-z (PMC5532403; doi:10.1007/s00280-017-3372-z)
Supplement: Supplementary file 1 — Supplemental Fig. 1 Correlation of pulse measured by machine or manual counts. Pulse was quantified via machine (indirect blood pressure monitor) and manual counting (palpation or auscultation). There was a strong correlation (0.8973) between pulse quantified with the two methods. Pulse was quantified via manual counting for the multiple dosing experiment. Supplemental Fig. 2 Variations in indirect blood pressure following administration of single dose of MnBuOE. Subcutaneous administration of MnBuOE caused subclinical fluctuations in recorded indirect blood pressures. Administration of MnBuOE at 0.25mg/kg resulted in the most stable maintenance of initial blood pressures. Supplemental Fig. 3 Variations in heart rate and blood pressure following administration of single dose of MnBuOE. Dog 2 and Dog 3 were treated with diphenhydramine (2 mg/kg intramuscularly) upon the first evidence of anaphylactic drug reaction (20 minutes post-injection MnBuOE). Dog 3 was treated with 3 intravenous boluses of lactated ringer’s solution (10–12 mL/kg over 15 minutes). Tachycardia persisted following treatment with diphenhydramine (n = 2/2) and intravenous fluid therapy (n= 1/1). The effects of treatment with diphenhydramine and/or intravenous fluid therapy on indirect blood pressure measurements are unclear (PDF 165 kb) [file 280_2017_3372_MOESM1_ESM.pdf]

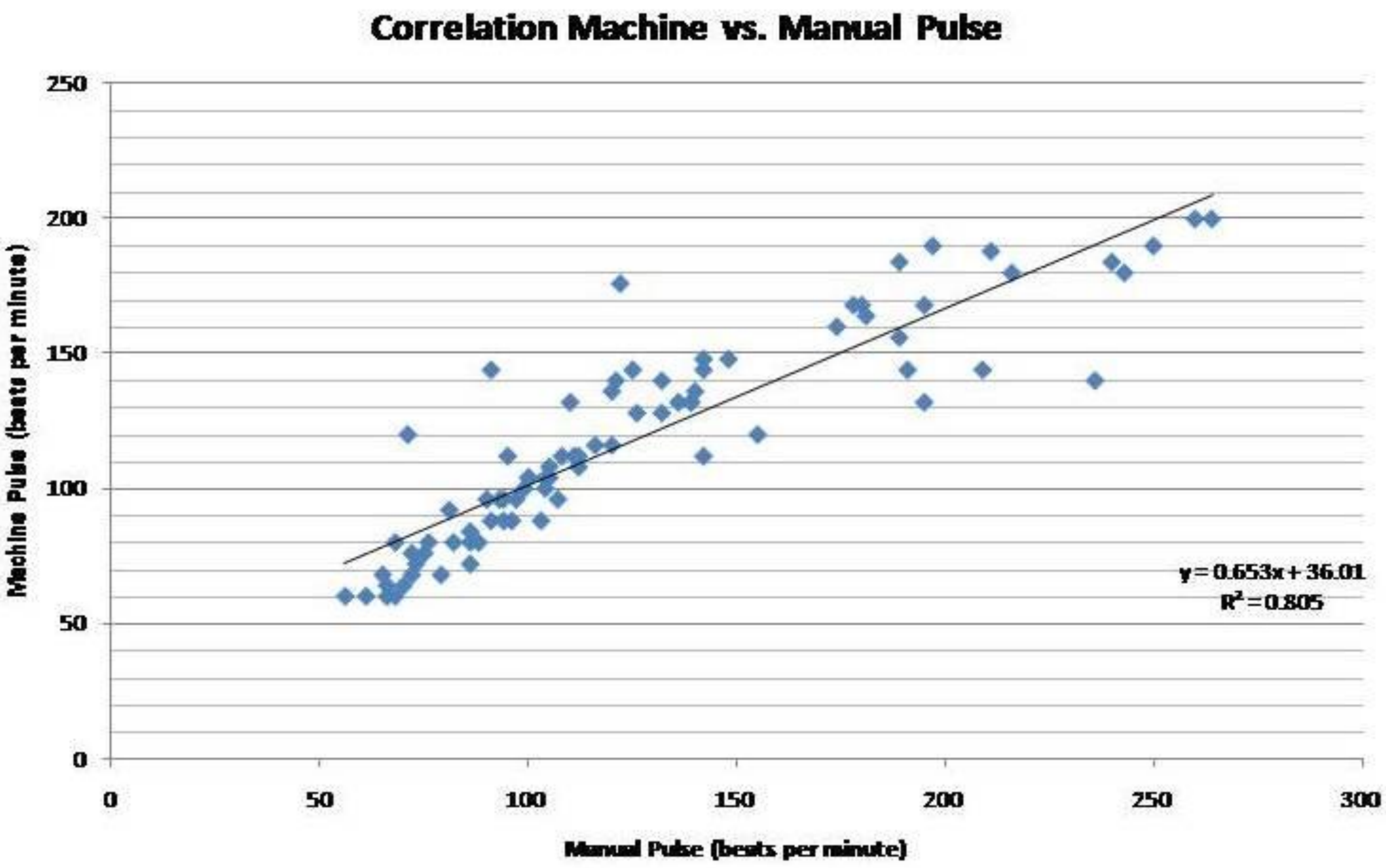

Correlation Machine vs. Manual Pulse
Machine Pulse (beats per minute)
Manual Pulse (beats per minute)
0
50
100
150
200
250
300
y = 0.653x + 36.01
R² = 0.805

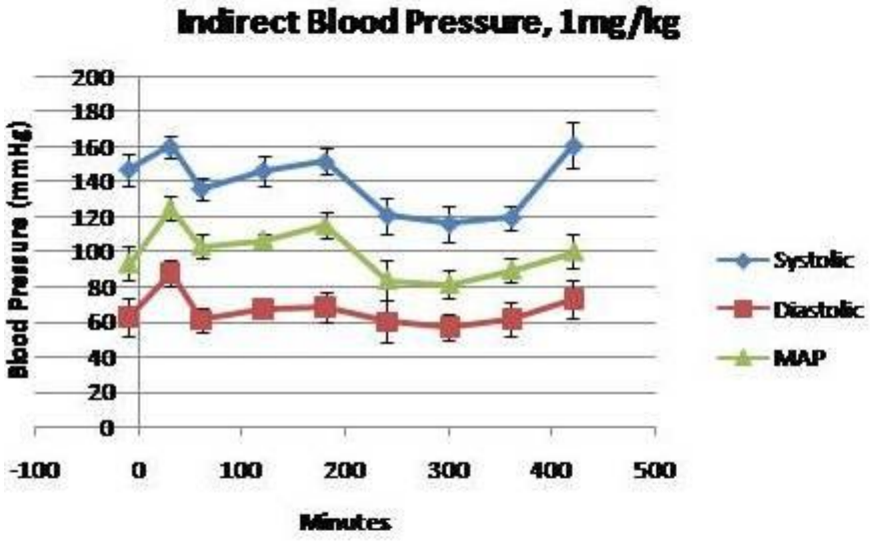
Indirect Blood Pressure, 1mg/kg
Blood Pressure (mmHg)
Minutes
Systolic
Diastolic
MAP

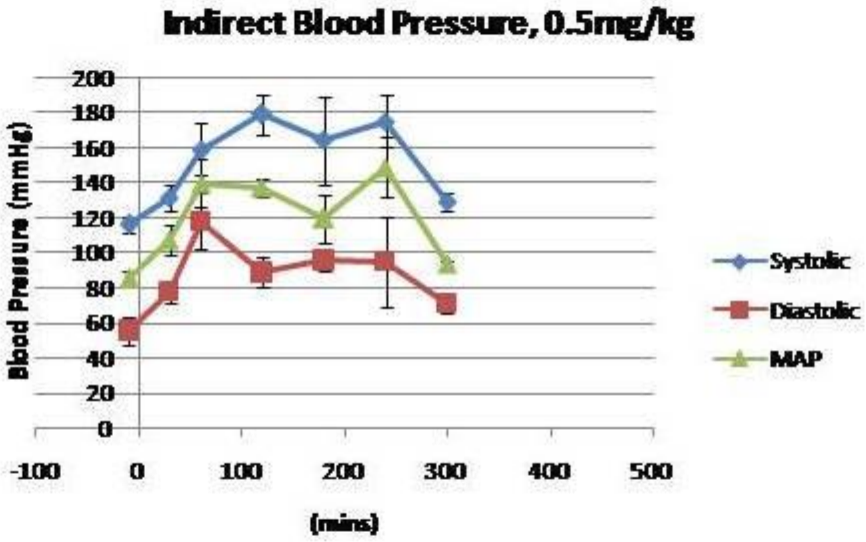
Indirect Blood Pressure, 0.5mg/kg
Blood Pressure (mmHg)
(mins)
Systolic
Diastolic
MAP

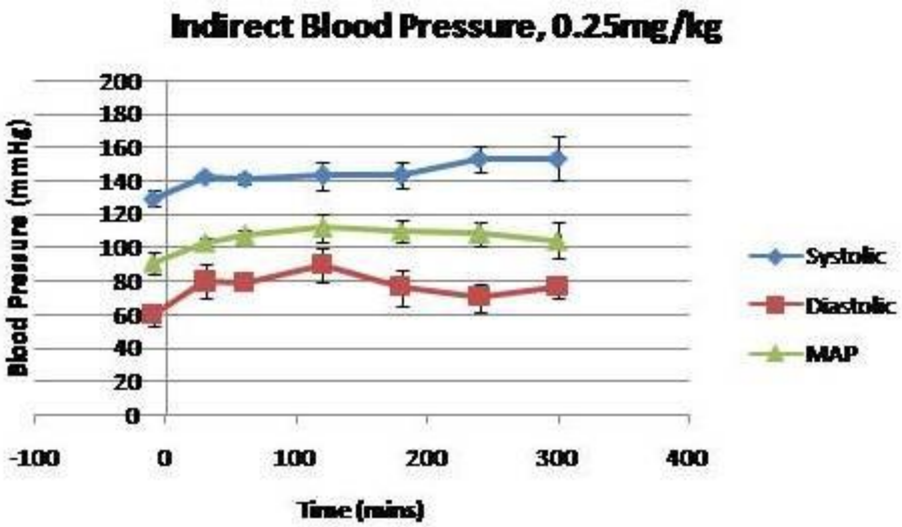
Indirect Blood Pressure, 0.25mg/kg
Blood Pressure (mmHg)
Time (mins)
Systolic
Diastolic
MAP

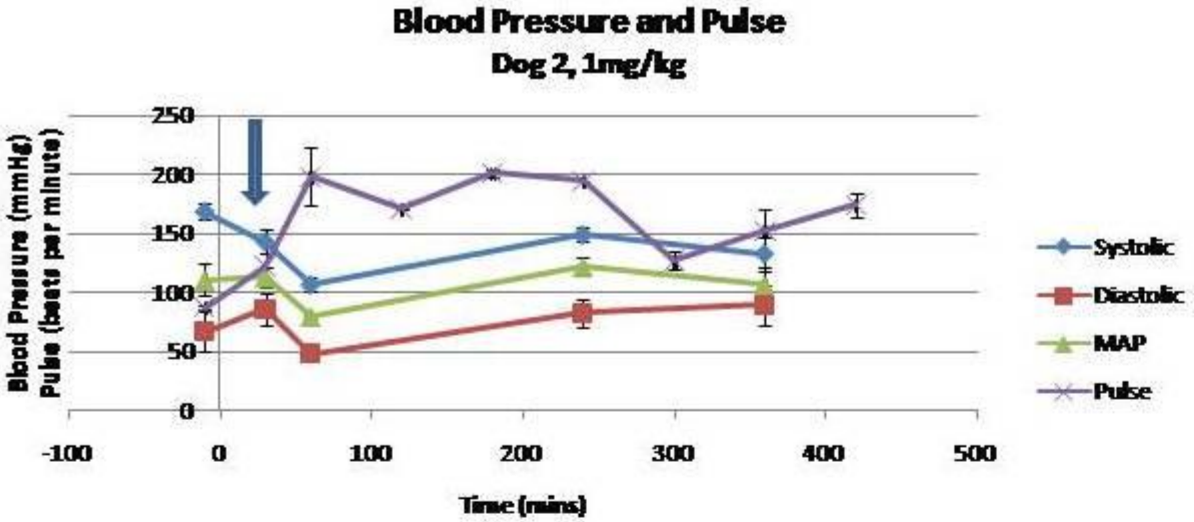
Blood Pressure and Pulse
Dog 2, 1mg/kg
Blood Pressure (mmHg)
Pulse (beats per minute)
250
200
150
100
50
0
-100
0
100
200
300
400
500
Time (mins)
Systolic
Diastolic
MAP
Pulse

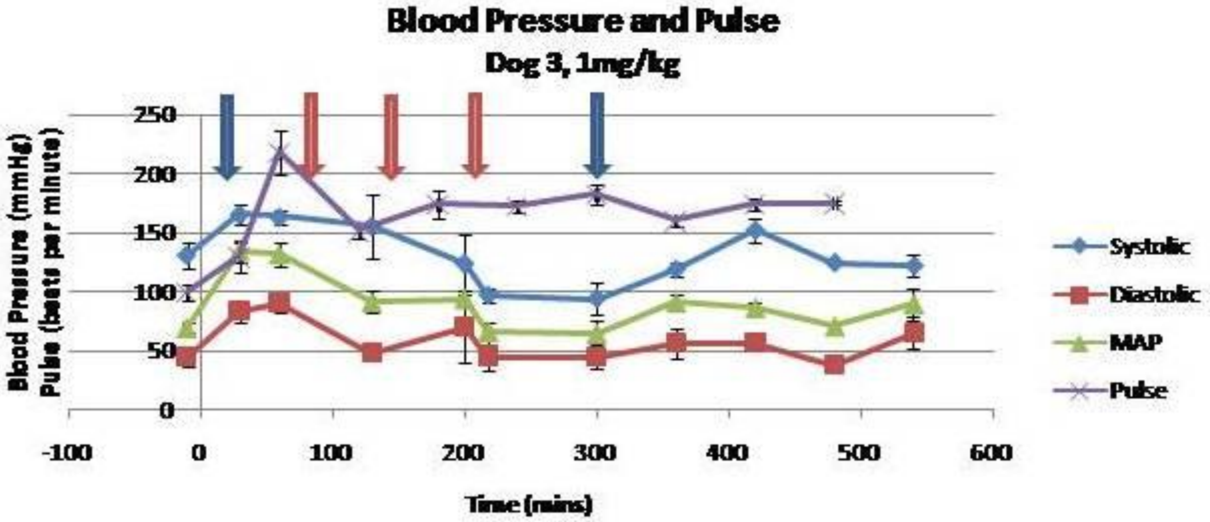
Blood Pressure and Pulse
Dog 3, 1mg/kg
Blood Pressure (mmHg)
Pulse (beats per minute)
250
200
150
100
50
0
-100
0
100
200
300
400
500
600
Time (mins)
Systolic
Diastolic
MAP
Pulse
